# Activity of a Carboxyl-Terminal Truncated Form of Catechol 2,3-Dioxygenase from *Planococcus* sp. S5

**DOI:** 10.1155/2014/598518

**Published:** 2014-02-13

**Authors:** Katarzyna Hupert-Kocurek, Danuta Wojcieszyńska, Urszula Guzik

**Affiliations:** Department of Biochemistry, Faculty of Biology and Environment Protection, University of Silesia in Katowice, Jagiellonska 28, 40-032 Katowice, Poland

## Abstract

Catechol 2,3-dioxygenases (C23Os, E.C.1.13.12.2) are two domain enzymes that catalyze degradation of monoaromatic hydrocarbons. The catalytically active C-domain of all known C23Os comprises ferrous ion ligands as well as residues forming active site pocket. The aim of this work was to examine and discuss the effect of nonsense mutation at position 289 on the activity of catechol 2,3-dioxygenase from *Planococcus* strain. Although the mutant C23O showed the same optimal temperature for activity as the wild-type protein (35°C), it exhibited activity slightly more tolerant to alkaline pH. Mutant enzyme exhibited also higher affinity to catechol as a substrate. Its *K*_*m*_ (66.17 *µ*M) was approximately 30% lower than that of wild-type enzyme. Interestingly, removal of the C-terminal residues resulted in 1.5- to 1.8-fold (*P* < 0.05) increase in the activity of C23OB61 against 4-methylcatechol and 4-chlorocatechol, respectively, while towards catechol the activity of the protein dropped to about 80% of that of the wild-type enzyme. The results obtained may facilitate the engineering of the C23O for application in the bioremediation of polluted areas.

## 1. Introduction

Aromatic compounds enter the environment either through natural processes (degradation of plant and animal residues) or as the result of anthropogenic activities [1]. They are common constituents of wastes originating from chemical, pharmaceutical, explosive, dyes, and agrochemicals industry [2]. Due to their potential toxicity to living organisms, including humans, as well as their mutagenicity and carcinogenicity aromatic compounds are pollutants of great environmental concern [2, 3]. Although aromatic molecules are considered to be among the most difficult to remove most of them are used by bacterial species as growth substrates [4–6]. The ability of microorganisms to grow in the presence of such compounds enables their potential applications for bioremediation strategies.

The strategy for degradation of aromatic structure by aerobic bacteria comprises hydroxylation and cleavage of the aromatic ring of catechol and its derivatives with the usage of oxygenases [7, 8]. One of the major mechanisms for aromatic ring degradation is its *meta*-fission catalyzed by extradiol dioxygenases in a position adjacent to the hydroxyl substituents, in a reaction involving incorporation of two atoms of oxygen [7, 9–11]. The catalytic mechanism of extradiol catechol dioxygenases is carried through several enzyme-bound intermediates. The reaction starts with bidentate binding of the substrate as catecholate monoanion to the active-site metal with simultaneous displacing of two water molecules [12, 13]. Binding of the substrate increases the affinity of the metal center for O_2_ binding. The oxygen binds to the ferrous form of the enzyme resulting in delocalization of electron density onto the bound O_2_ and semiquinone-FeII-superoxide intermediate formation. Subsequent Criegee rearrangement and O–O bond cleavage give a lactone intermediate and an FeII-bound hydroxide ion. Hydrolysis of the lactone and release of the reaction product (2-hydroxymuconate semialdehyde) complete the reaction cycle [7, 13].

Extradiol dioxygenases are versatile enzymes reacting with a wide range of substrates. They can be divided into two separate clades: those showing preference for monoaromatic compounds and those showing preference for polycyclic substrates [14]. Catechol 2,3-dioxygenases (C23Os) belonging to the extradiol dioxygenases family are involved in monoaromatic compounds degradation. These two domain enzymes are mainly oligomers and utilize a nonheme ferrous ion, and rarely manganese (II) or magnesium (II), to cleave the aromatic ring [7, 15, 16]. The catalytic metal in the active site is located at the end of a long funnel of the C-terminal domain. It is coordinated by three well-conserved residues, two histidines and one glutamate forming 2-His-1-carboxylate facial triad motif [12, 13]. Because of their importance in degradation of aromatic ring, C23Os have received in last decades a lot of attention. Knowledge of the molecular determinants relevant for their function and substrate specificity is essential to widen the ability of the microorganisms to transform aromatic compounds [17, 18].

C23O is catechol 2,3-dioxygenase that catalyzes ring cleavage of the catecholic compounds in Gram-positive *Planococcus* sp. strain S5, which is able to utilize benzoate, hydroxybenzoate, dihydroxybenzoate, phenol, and salicylate as growth substrates [19–21]. The structural gene for this enzyme (GenBank ID: **HQ223337.2**) was found in the plasmid and the nucleotide sequencing, and homology search revealed that it shares the greatest homology with isoenzymes from Gram-negative *Pseudomonas* strains [20]. Additionally, the product analysis and its sequence alignment with other dioxygenases suggest that C23O from strain S5 belongs to the type I superfamily of extradiol dioxygenases [22].

In our previous work, *c23o* gene from *Planococcus *sp. S5 was cloned and randomly mutated using error-prone PCR. From a random library of mutants 8 clones carrying at least one nonsilent mutation were obtained. In one of those mutant form of catechol 2,3-dioxygenase TGC codon for cysteine at position 103 had been changed to a CGC codon for arginine and the AAG codon at position 289 for lysine had been changed to a UAG stop codon [22]. While Cys103Arg amino acid substitution is localized within inactive N-terminal domain, Lys289stop substitution resulting in shortened protein is localized within C-domain of the enzyme [22]. As the C-terminal domain of all known C23Os is catalytically active [17, 23] the aim of the present study was to determine the effect of removal of terminal amino acid residues on catechol 2,3-dioxygenase structure and activity. The wild-type enzyme (C23Owt) and its carboxyl-terminal truncated form (C23OB61) were functionally expressed in *E. coli* and their activity and substrate specificity were characterized. Additionally, a putative three-dimensional (3D) structure of wild-type and mutant enzyme was determined. The significance of this study is to discuss the variation in activity of the wild-type and mutated catechol 2,3-dioxygenase towards methylcatechol- and 4-chlorocatechol based on the differences in structural properties of examined proteins. This knowledge gives clues to the design of enzymes with higher activity and altering substrate specificity.

## 2. Materials and Methods

### 2.1. Bacterial Strains and Growth Conditions

The bacterial strains and plasmids used in this study are listed in Table 1. Cells of *Escherichia coli* BL21 were grown in LB medium at 37°C and agitated at 125 rpm. All other strains harboring plasmids bearing the *c23o* gene were cultivated in LB medium supplemented with 100 *μ*g/mL ampicillin at 37°C at 125 rpm. For overexpression of the wild-type and mutant C23O 1 mM isopropyl-*β*-D-thiogalactopyranoside (IPTG) was added to the medium.

### 2.2. Subcloning of *c23oB61* Gene

The recombinant pUC19*c23oB61* plasmid carrying mutated *c23o* gene was isolated from DH5C23OB61 clone with Plasmid Mini Kit (A&A Biotechnology, Poland) and digested with *EcoR*I and *Hind*III endonucleases (Fermentas) by standard procedures [24]. After gel purification, the DNA carrying the mutagenized gene was ligated into the *EcoR*I and *Hind*III endonuclease sites of pET-22(b) with T4 DNA ligase (Fermentas). The ligation mixture was then transformed into competent *E. coli* BL21 cells and plated on LB medium supplemented with ampicillin (100 *μ*g/mL). Transformation of *E.coli* with plasmid DNA was performed by the RbCl procedure [25]. From transformants plasmids were isolated and those containing the correct insert were identified by restriction enzyme analysis. The presence of mutations in *c23oB61 *gene was then confirmed by DNA sequencing.

### 2.3. DNA Sequencing and Analysis

DNA sequencing was performed at the Department of Molecular Biology, Institute of Oncology DNA Sequencing Facility by using Big Dye_Terminator Cycle Sequencing Kit (Applied Biosystem) and AbiPrism_3100 Genetic Analyzer. Assembly and analysis of DNA sequences were performed with Chromas LITE software (Technelysium Pty, Tewantin, Australia).

### 2.4. Molecular Modeling of Mutant and the Wild-Type Catechol 2,3-Dioxygenase

Amino acid sequence of mutated form and the wild-type catechol 2,3-dioxygenase was deduced using the CLC Free Workbench 4.0.1 software. The deduced structure of the enzymes was modeled using the interactive mode of the 3D-JIGSAW protein comparative modeling server (http://bmm.cancerresearchuk.org/~3djigsaw/). Structure models as x.pdb data files were analyzed using RasMol 2.6 software package.

### 2.5. Overexpression of C23O in *E. coli* and Preparation of Cell-Free Extract

For overexpression of C23O, bacterial strains BL21C23Owt and BL21C23OB61 harboring *c23owt* and *c23oB61m*, respectively, were cultivated in 50 mL of LB medium supplemented with ampicillin (100 *μ*g/mL) overnight at 37°C, and 5 mL of cells was then subcultured into 250 mL of the same medium and cultured under the same conditions. After the cell OD600 reached 0.4–0.6, IPTG was added to the final concentration of 1 mM. After further incubation for 4 h, cells were centrifuged at 4500 ×g for 15 min at 4°C. Next, the cells were washed with 50 mM phosphate buffer, pH 7.5, resuspended in the same buffer at concentration equivalent to an OD600 of 1.0, and ruptured by pulsed sonication (Vibra Cell TM) 6 times for 15 s. The disrupted cells suspensions were centrifuged at 9000 ×g for 30 min at 4°C to remove cell debris. The clear supernatant was used as crude extract for enzyme assays.

### 2.6. Enzyme Assay

In order to determine catechol 2,3-dioxygenase activity, the formation of 2-hydroxymuconic semialdehyde (2-HMS) was measured at 375 nm (*ε*_2-HMS  at  375 nm_ = 36,000/M cm) in a reaction mixture containing 20 *μ*L of catechol (50 mM), 960 *μ*L of phosphate buffer pH 7.5 (50 mM), and 20 *μ*L of crude extract in a total volume of 1 mL [26]. Control reactions (without crude extract) were performed for each assay. Protein concentrations of the crude extract from cultured bacteria were determined by the Bradford method [27]. One unit of C23O activity was defined as the enzyme amount required generating 1 *μ*mol of product per minute. The specific activity is defined in mU per milligram of protein.

### 2.7. The Effect of pH and Temperature on C23O

The effect of pH on the enzymatic activity of mutant and the wild-type catechol 2,3-dioxygenase was determined by measuring the activity at 35°C, over the pH range of 2.2–10.7 using the following: 0.05 M glycine buffer (pH 2.2), 0.05 M phosphate-citrate buffer (pH 3.0 to 5.0), 0.05 M Sörensen-phosphate buffer (pH 6.0 to 8.0), 0.05 M borate buffer (pH 9.0), and carbonate buffer (pH 9.2 to 10.7).

The effect of temperature on the enzymatic activity of mutant and the wild-type enzyme was determined by measuring the activity using the reaction system described above with temperatures ranging between 4 and 50°C. All experiments were performed in 50 mM Tris-HCl buffer (pH 7.0). The enzyme and substrate solutions were preincubated and mixed and the enzymatic reaction was then carried out at the same temperature.

### 2.8. Determination of Kinetic Constants of C23O

The catalytic parameters (Michaelis-Menten constant, *K*_*m*_, and maximum velocity, *V*_max⁡_) were calculated for the wild-type and mutated form of C23O by measuring initial linear rates of the enzymatic reaction after the addition of different concentrations of the catechol ranging from 0 to 150 *μ*M at 35°C. At least four independent measurements were carried out for each substrate concentration. *K*_*m*_ and *V*_max⁡_ were calculated from the Hill equation using nonlinear regression analysis using the SigmaPlot software (SigmaPlot, version 12.0).

### 2.9. Substrate Specificity of C23O

The substrate specificity of mutated and wild-type catechol 2,3-dioxygenase was examined with 3-methylcatechol, 4-methylcatechol, and 4-chlorocatechol. The aromatic ring cleavage was determined by measuring the increase in the absorbance at the corresponding wavelength of each *meta*-cleavage product formed. Activity of C23O was assayed under the reaction conditions described above, using tested aromatic compounds instead of catechol as a substrate. The molar extinction coefficient used for the product from 3-methylcatechol was 13,800/M cm (at 388 nm), from 4-methylcatechol was 28,100/M cm (at 382 nm) [28], and from 4-chlorocatechol 40,000/M cm (at 379 nm) [29].

### 2.10. Statistical Analysis

All experiments were performed in four replicates. The obtained data were analyzed by one-way ANOVA and the statistical significance of differences in measured data was assessed by a post hoc comparison of the means using the least significant differences (LSD) test. Statistical analysis was performed using STATISTICA 10.0 PL software package.

## 3. Results and Discussion

### 3.1. Structural Properties of the Wild-Type and Mutant Catechol 2,3-Dioxygenase

Amino acid sequence in the polypeptide chain defines protein structure and properties. In order to determine the effect of mutations (C103R, K289Stop) on the structure and activity of catechol 2,3-dioxygenase from *Planococcus* strain, the 918-bp insert encoding carboxyl-terminal truncated form of C23O in the clone DH5C23OB61 [22] was subcloned into pET-22(b) vector to express the mutated enzyme in *E. coli *BL21. Localization of mutations was then confirmed by sequence analysis and amino acid sequence of the mutant enzyme was deduced. Based on the deduced amino acid sequence by using the interactive mode of the 3D-JIGSAW protein comparative modeling server the 3D structure of the wild-type and mutant form of catechol 2,3-dioxygenase from *Planococcus* strain was predicted.

**Figure 1:**
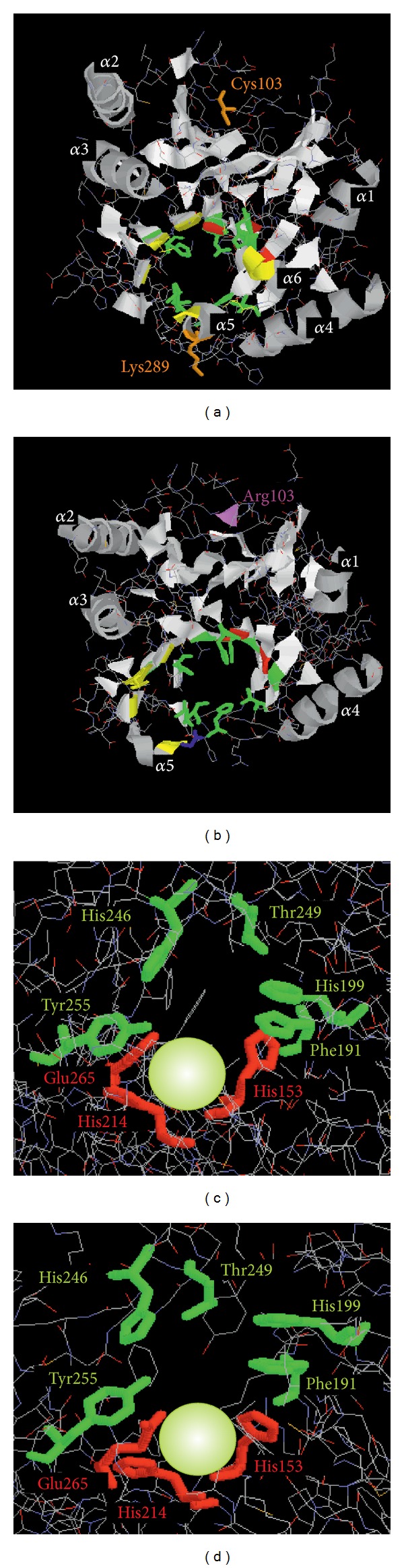
The deduced 3D structure of the wild-type and mutated C23O from *Planococcus* sp. S5. Top row localization of the molecular domains of wild-type (a) and mutant (b) protein. Bottom row predicted structure of the active sites of the wild-type (c) and mutant (d) protein. Fe atoms are shown as light green spheres; the three residues involved in ferrous ions coordination are shown in red and amino acid residues involved in substrate binding are shown in green. Amino acid residues forming the entrance to the active site pocket are shown in yellow.

Molecular studies on three-dimensional structure of catechol 2,3-dioxygenase (MPC) from *Pseudomonas putida* mt-2 have shown that the active site of the enzyme is located in the cavity within the C-terminal domain. The cavity is arranged as a funnel that is open to the molecular surface from one site and to an intersubunit interface from the other end. C-terminal end region of the C-terminal domain consists of 27 amino acid residues (Val280-Thr307) that cover and narrow the open region of the funnel [30]. In our studies structure of *P. putida* mt-2 catechol 2,3-dioxygenase (PDB accession code: 1mpy [30]) served as template for homology modeling of the wild-type and mutant C23O. Our data (Figures 1(a) and 1(b)) suggests the presence of externally located *α*-helices and internally located *β*-sheets, structures typical for many extradiol dioxygenases [16, 31, 32]. Additionally, the amino acids involved in metal binding in C23O from *Planococcus* strain (H153, H214 and E265) (Figures 1(c) and 1(d)) are the same as those of XylE and other extradiol dioxygenases [30–32]. The model of C23Owt (Figure 1(a)) shows 6 *α*-helices (*α*1–*α*6) analogous to the XylE model [30], while in the mutant form of protein (Figure 1(b)), as a result of nonsense mutation at position 289, *α*6 is removed and *α*5 is inclined that results in wider entrance to the active site.

### 3.2. Effect of pH on the Mutant Catechol 2,3-Dioxygenase

**Figure 2:**
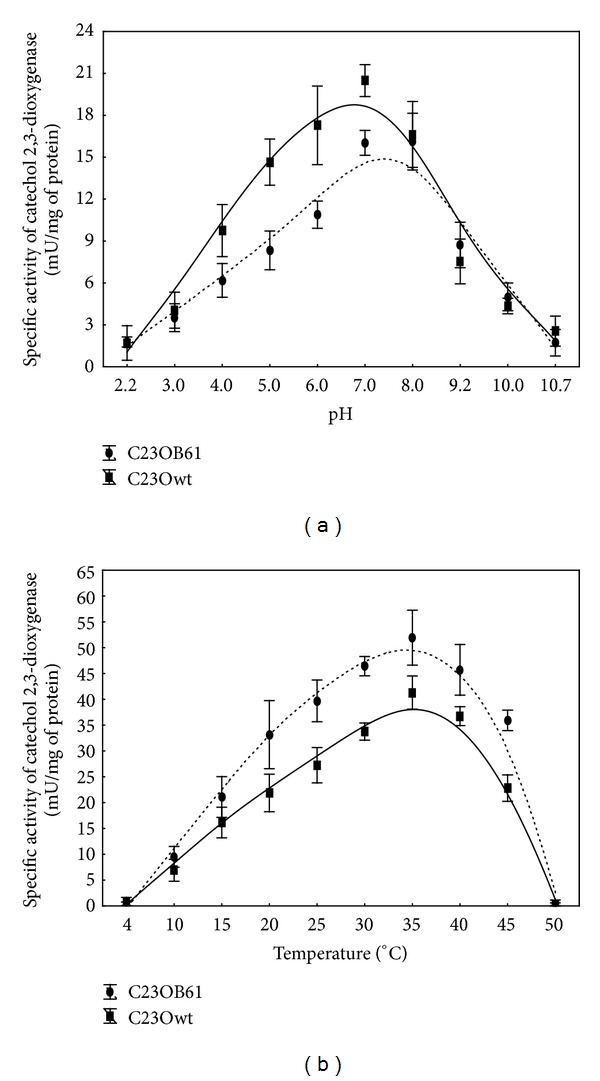
Effects of pH (a) and temperature (b) on the activity of wild-type (C23Owt) and the mutant (C23OB61) dioxygenases. The data shown in this figure represent the mean ± SD of four experimental replicates.

To verify if introduced mutations influenced C23O activity, the effect of pH on the mutated and wild-type enzyme activity was determined. The wild-type C23O showed the highest activity at pH 7.0, while C23OB61 mutant exhibited activity slightly more tolerant to alkaline pH (Figure 2(a)) and was still 60% active at pH 9.2. pH between 7.0 and 7.5 was optimal for the wild-type C23Os from thermoacidophilic *Sulfolobus solfataricus* strain 98/2 [33], *Bacillus thermoleovorans* A2 [34], and rubber degrading bacterium *Gordonia polyisoprenivorans* [35]. Optimum pH at 8.0 showed catechol 2,3-dioxgenase from *Stenotrophomonas maltophilia* KB2 [36], while C23O from *Pseudomonas putida* GJ31 was the most active at pH 9.6. C23Os isolated from *Bacillus* strains [6] showed the highest activity at pH 10.0–11.0. Wild-type ThnC, an extradiol dioxygenase of a I.3 family involved in tetralin biodegradation, showed a strong dependence on pH with an optimum at pH 6.8–7 and just 35–40* *% of the activity at pH 5.5. Some mutants of this enzyme (N213H, G206 M, A282R, and A282G) exhibited activity significantly more tolerant to acidic pH, although their pH optima were the same as that of wild-type ThnC, while for mutant optimal pH was displaced from 7.0 to 5.5 [9]. Substitution of arginine at position 296 with glutamine shifted an optimum pH of catechol 2,3-dioxygenase from *Planococcus* strain from 7.0 to 5.0. Activity of this mutant protein at pH 5.0 and pH 4.0 relative to its activity at pH 7.0 was 144.7% and 124%, respectively [22]. In contrast, mutated C23O from *Pseudomonas* strain became more alkalescency stable compared with the wild-type enzyme and retained 75% of the maximal enzyme activity even under pH 9.5 [37].

### 3.3. Effect of Temperature on the Mutant Catechol 2,3-Dioxygenase

Most of the examined C23Os show maximal activity at 30–40°C [35, 37–39]. The temperature optimum of the reaction rate of catechol 2,3-dioxygenase from *Pseudomonas putida* GJ31 was estimated to be 50°C. However, denaturation of the enzyme was significant at this temperature [40]. C23O isolated from thermophilic *Bacillus thermoleovorans* A2 [34] as well as four other *Bacillus* isolates [6] showed the highest activity at 70°C. However, the half-life of the enzyme from strain A2 was only 1.5 min at this temperature [34]. It has been shown that introduced mutations may affect thermostability of enzymes. For example, replacement of Ala229 and His294 in catechol-2,3-dioxygenase from *Pseudomonas* sp. CGMCC2953 by cysteine resulting in formation of additional disulfide bond widened the optimum temperature of such mutated enzyme from 40°C to 40–50°C [37]. The proline rule for thermostabilizing proteins has been proposed by Suzuki et al. [41]. For different enzymes, including extradiol dioxygenases, the percentage of Pro residues was found to be higher in thermostable compared to mesophilic ones [42, 43]. In molecular stabilization of protein introduction of prolines was effective for *α*-glucosidase from *Thermoanaerobacter tengcongensis* MB4 or methyl parathion hydrolase from *Ochrobactrum* sp. M231 [44, 45]. Determination of the effect of temperature on the mutated and wild-type catechol 2,3-dioxygenase activity indicated that introduced mutations did not change optimal temperature for the enzyme activity. Either C23Owt or C23OB61 showed the highest activity at 35°C (Figure 2(b)). Analysis of amino acid sequences of C23OB61 mutant has shown that introduced mutations did not increase the percentage of cysteine and proline residues. We may speculate that for this reason temperature profiles of the mutant and wild-type enzyme activity were similar.

### 3.4. Catalytic Parameters of the Mutant Protein

**Figure 3:**
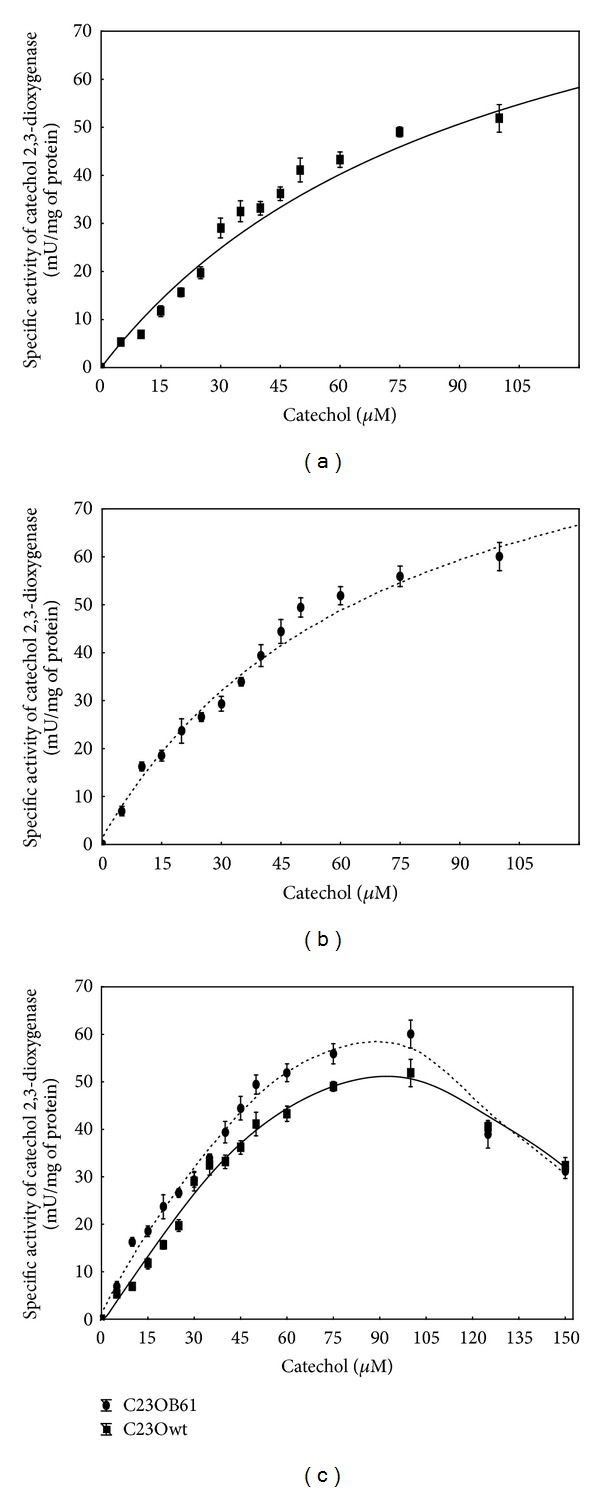
Effects of substrate concentration on the activity of wild-type ((a), (c)) and mutant C23OB61 ((b), (c)) protein. The data points represent the mean ± SD of four experimental replicates.

In order to investigate the effect of introduced mutations on the C23O activity for catechol, the specific activities of wild-type and mutant enzyme for this substrate were measured using recombinant *E. coli* extracts (Table 2). Using various concentrations of catechol as the substrate *K*_*m*_ and *V*_max⁡_ of wild-type C23O and its mutant were determined (Table 3). Although C23OB61 showed lower activity towards catechol than the wild-type enzyme (Table 2), it exhibited higher affinity to this substrate (Table 3). Its *K*_*m*_ (66.17 ± 9.44 *μ*M) was approximately 30% lower than that of wild-type enzyme (97.74 ± 10.91 *μ*M) (Figure 3). Based on the predicted structures (Figure 1), we can hypothesize that obtained mutations, especially removal of residues involved in formation of the lid over the substrate-binding pocket, provide an easier access of the substrate to the active site and for positioning the catechol molecule bound to iron. According to Riegert et al. [46], a reduced accessibility of the active center to the substrate reduced substrate affinity of the mutant form of 2,3-dihydroxybiphenyl 1,2-dioxygenase from *Sphingomonas xenophaga* BN6.

### 3.5. Substrate Range of the Mutant Protein

Extradiol dioxygenases cleave a wide variety of catecholic and noncatecholic substrates [17]. From this group of enzymes, catechol 2,3-dioxygenases are known to catalyze ring cleavage of catechol and its chloro-, methyl-, and ethyl-derivatives [5, 12, 47]. However, the reactivity of C23Os to catechol is usually higher than the reactivity of these enzymes to substituted catechols [5, 47]. It has been suggested that substituted catechols promote more rapid inactivation of the enzyme because the substituent hinders the correct positioning of the substrate or of dioxygen at the active site [46, 48]. In this study the substrate range of C23Owt and mutant protein was examined by incubating the enzyme with catechol, 3-methylcatechol, 4-methylcatechol, or 4-chlorocatechol and determining the rate of appearance of products. Interestingly, the obtained mutations caused an increase in the activity of C23O against *para*-substituted catechol derivatives, while towards catechol the activity of mutant protein dropped to about 80% of that of the wild-type enzyme (Table 2). Considering that the C-terminal region of mutant protein is shorter compared with that from wild-type C23O there is a wider entrance to the active site in C23OB61 compared with C23Owt. This in turn facilitates the access and proper positioning of the substrates with chloro- and methyl-groups in the mutant enzyme active site. The role of C-terminal tail in determination of substrate specificity of AkbC by restricting the access of large substrates to the active site was also suggested by Cho et al. [12]. After removal of the last five of 305 amino acid residues of AkbC they observed 7-fold increase in the enzyme activity against 3-methylcatechol and even 9-fold increase in AkbC activity towards dihydroxybenzoate. Although for C23O enzymatic activity the C-terminal domain is essential, we cannot exclude the possibility that mutation C103R within N-domain of C23O may also affect the enzyme parameters. However, this speculation needs further investigations.

## 4. Conclusions

We showed that substrate specificity and activity of catechol 2,3-dioxygenase can be modulated by shortening the protein at C-terminal end. The removal of terminal amino acid residues forming the lid that covers the entrance to the substrate-binding pocket of C23O caused an increase in activity of the enzyme against 4-methylcatechol and 4-chlorocatechol. Obtained results may facilitate the engineering of the C23O for bioremediation of environments contaminated with cresols and chloroaromatic compounds.

## Figures and Tables

**Table 1:** Strains and plasmids.

Strain or plasmid	Genotype and/or properties (reference); source
DH5C23OB61	*E. coli D* *H*5*α*[*F* ^−^ * endA1 glnV44 thi-1 recA1 relA1 gyrA96 deoR nupG *Φ*80dlacZΔM15 Δ(lacZYA-argF)U169, hsdR17( r*_*K*_^−^ *m*_*K*_^+^ *), λ*–] harboring *c23oB61* gene [22]; laboratory stock

*E. coli* BL21	[*fhuA2 [lon] ompT gal [dcm] ΔhsdS*]; New England Biolabs

BL21C23Owt	*E. coli* BL21 harboring c*23owt *gene [22]; laboratory stock

BL21C23OB61	*E. coli* BL21 harboring *c23oB61* gene (this work)

pUC19* c23oB61 *	Amp^r^, pUC19 derivative, *c23oB61* encoding mutant form of catechol 2,3-dioxygenase (C103R, K289Stop) [22]

pET-22(b)	Amp^r^; Novagen

p*c23oB61 *	Amp^r^, pET-22(b) derivative, *c23oB61* encoding mutant form of catechol 2,3-dioxygenase (C103R, K289Stop) (this work)

**Table 2:** Specific and relative activity of wild-type (wt) and mutant C23O dioxygenases using different catechols as the substrate.

Substrate	C23Owt			C23OB61		
	Specific activity (mU/mg)^a^	Relative activity^b^ %	Relative activity^c^ %	Specific activity (mU/mg)^a^	Relative activity^b^ %	Relative activity^c^ %
Catechol	20.90 ± 0.94*	100	100	16.45 ± 0.83	78.70	100
3-Methylcatechol	1.28 ± 0.55	100	6.12	2.33 ± 1.09	182.03	14.16
4-Methylcatechol	21.30 ± 1.56	100	101.91	30.98 ± 3.74*	145.45	188.32
4-Chlorocatechol	9.38 ± 2.48	100	44.88	16.75 ± 1.76*	178.57	101.82

*P < 0.05. ^a^One unit of C23O activity was defined as the enzyme amount required generating 1 µmol of product per minute. The specific activity is defined in mU per milligram of protein.

^b^Expressed as a percentage of the C23Owt-specific activity which is set as 100%.

^c^Expressed as a percentage of the C23Os-specific activity toward catechol as a substrate which is set as 100%.

All data shown were expressed as mean ± standard deviation (*n* = 4).

**Table 3:** Kinetic parameters for catechol oxygenation to 2-hydroxymuconic semialdehyde by wild-type (wt) and mutant C23O dioxygenases.

Enzyme/substrate	C23Owt		C23OB61	
	*K*_*m*_ (*µ*M)	*V*_max⁡_ (mU/mg)	*K*_*m*_ (*µ*M)	*V*_max⁡_ (mU/mg)
Catechol	97.74 ± 10.91	115.38 ± 14.40	66.17 ± 9.44	104.50 ± 8.43

All data shown were expressed as mean ± standard deviation (*n* = 4).
